# A myxobacterial GH19 lysozyme with bacteriolytic activity on both Gram-positive and negative phytopathogens

**DOI:** 10.1186/s13568-022-01393-y

**Published:** 2022-05-12

**Authors:** Yueqiu Li, Xiaoli Zhou, Xianjiao Zhang, Zhiqiang Xu, Honghong Dong, Guohui Yu, Ping Cheng, Qing Yao, Honghui Zhu

**Affiliations:** 1 Key Laboratory of Agricultural Microbiomics and Precision Application-Ministry of Agriculture and Rural Affairs, Guangdong Provincial Key Laboratory of Microbial Culture Collection and Application, State Key Laboratory of Applied Microbiology Southern China, Guangdong Microbial Culture Collection Center (GDMCC), Institute of Microbiology, Guangdong Academy of Sciences, Guangzhou, 510070 China; 2 Key Laboratory of Green Prevention and Control on Fruits and Vegetables in South China, Ministry of Agriculture and Rural Affairs, Guangdong University Key Laboratory for Sustainable Control of Fruit and Vegetable Diseases and Pests, Innovative Institute for Plant Health, College of Agriculture and Biology, Zhongkai University of Agriculture and Engineering, Guangzhou, 510225 China; 3 Center for Litchi, Guangdong Province Key Laboratory of Microbial Signals and Disease Control, Guangdong Engineering Research Center for Grass Science, Guangdong Engineering, College of Horticulture, South China Agricultural University, Guangzhou, 510642 China

**Keywords:** Myxobacteria, Lysozyme, Bacteriolytic activity, Phytopathogens

## Abstract

Myxobacteria, as predatory bacteria, have good application potential in the biocontrol of pathogenic microorganisms. Extracellular enzymes are thought to play an important role in their predation and also provide resources for discovering new antibacterial molecules. We previously isolated a myxobacterium, *Corallococcus silvisoli* c25j21 GDMCC 1.1387, which is predatory to plant pathogenic bacteria. In this study, we identified an endolysin-like GH19 glycoside hydrolase, C25GH19B, from the genome of c25j21. After its heterologous expression and purification from *E. coli*, the enzymatic properties of C25GH19B were characterized. C25GH19B showed lysozyme activity with the optimal reaction conditions at 40 °C and pH 4.5–5.0. Moreover, C25GH19B showed bacteriolytic activity against both Gram-positive and Gram-negative plant pathogenic bacteria. Our research provides not only a candidate enzyme for the development of novel biocontrol agents but also an experimental basis for further study on the function and mechanisms of extracellular enzymes in myxobacterial predation.

**Supplementary Information:**

The online version contains supplementary material available at 10.1186/s13568-022-01393-y.

## Introduction

As a promising alternative to chemical interventions, biocontrol of plant pathogens has the advantages of safety, eco-friendliness, and sustainability, and has a good application prospect in modern agriculture (Pirttila et al. 2021). Enzymes, such as plant and microbial chitinases, which have antifungal and insecticidal activities, have been reported as potential biocontrol agents against phytopathogenic fungi and insects e.g. *Fusarium roseum* and *Spodoptera litura* (Chandrasekaran et al. 2014; Chen et al. 2020; Gomaa 2021). While phytopathogenic bacteria such as *Pseudomonas* sp., *Curtobacterium* sp., etc. are the main pathogens causing crop infections. They spread in major crop production areas in the world and cause huge economic losses (Buttimer et al. 2017). The exploration and characterization of the microorganisms or enzymes with broad-spectrum lytic activity against phytopathogenic bacteria are necessary for the development of novel biocontrol strategies.

The glycoside hydrolase family 19 (GH19) includes chitinases from plants or bacteria, and lysozymes or endolysins from phages. Several bacteriophage-derived endolysins have been biochemically characterized and reported to have antimicrobial activity against Gram-positive or Gram-negative bacteria, and are considered to be ‘enzybiotics’ with good application potential (Orlando et al. 2020; Park et al. 2014). Among the characterized plant-derived GH19 enzymes, papaya chitinases exhibit bacteriolytic activities against *M. lysodeikticus* (Huet et al. 2006). However, there are few reports on the bacteriolytic activity of bacterial GH19 enzymes.

Predatory microorganisms, which kill other microorganisms and feed on them by secreting enzymes, provide us with a wealth of potentially valuable candidate enzyme resources. Myxobacteria are predators that are ubiquitous in the soil environment, lysing their prey and feeding on the released biomass (Munoz-Dorado et al. 2016). *Myxococcus xanthus* is the best-characterized myxobacterium and has been reported to prey on soil bacteria including plant pathogens, clinical pathogens, yeasts, and other fungi (Keane and Berleman 2016; Thiery and Kaimer 2020). In addition, other less explored myxobacteria such as *Corallococcus* sp., *Cystobacter* sp., *Sorangium* sp., and *Pyxidicoccus* sp., etc., also show predatory ability on microorganisms (Chambers et al. 2020; Livingstone et al. 2020, 2017, 2018).

Extracellular enzymes secreted by myxobacteria are thought to play an important role in their predation (Berleman and Kirby 2009; Konovalova et al. 2010; Sudo and Dworkin 1972). However, although myxobacteria prey on a wide variety of microorganisms, only a few extracellular enzymes have been characterized. Li et al*.* found that the outer membrane β-1,6-glucanase GluM and endo-chitinase CcCti1 are essential for *Corallococcus* sp. strain EGB to prey on phytopathogenic fungi *Magnaporthe oryzae* (Li et al. 2019a, 2019b). Arend et al*.* reported that a GH19 glycoside hydrolase, LlpM, from *M. xanthus* had lysozyme activity, and the secreted proteins of *M. xanthus* lysed Gram-positive bacteria e.g. *Micrococcus luteus* and *Bacillus subtilis*, but not Gram-negative bacteria (Arend et al. 2020). The diverse characteristics of bacterial cell walls require predators to evolve a variety of enzymes to lyse different types of prey. Myxobacterial enzymes with broad-spectrum bacteriolytic activity, especially against Gram-negative bacteria, remain to be further explored.

We previously isolated a myxobacterium strain, *Corallococcus silvisoli* c25j21 GDMCC 1.1387 (Zhang et al. 2022), which is predatory to bacteria and fungi. In this study, the predation of c25j21 on the phytopathogenic *C. flaccumfaciens* and *P. syringae* was investigated. An endolysin-like GH19 hydrolase (C25GH19B) in c25j21 genome was identified and expressed in *E. coli*. Furthermore, the enzyme activity, biochemical characteristics, and bacteriolytic activity against Gram-positive and negative phytopathogens were evaluated.

## Materials and methods

### Predation of c25j21 on *C. flaccumfaciens* and *P. syringae*

*C. silvisoli* c25j21 was inoculated on VY/2 agar plates (0.5% dried baker’s yeast, 0.1% CaCl_2_·2H_2_O, 1.5% agar) incubated at 30 °C for 5 days. Strain *Curtobacterium flaccumfaciens* GDMCC 1.343 and *Pseudomonas syringae* GDMCC 1.330 were inoculated in NB liquid medium and shaken at 160 rpm for 3 days at 30 °C. The freshly cultured pathogen was collected by centrifugation, washed, and resuspended in TPM buffer (10 mM Tris–HCl pH 7.6, 8 mM MgSO4, 1 mM KH2PO4) to adjust the optical density to OD_600_ = 5. Then a spot of 150 µL pathogen bacterial suspension was inoculated on the center of TPM agar (10 mM Tris–HCl-HCl pH 7.6, 8 mM MgSO4, 1 mM KH2PO4, 1.5% agar). A 5 mm diameter plug strain of c25j21 was inoculated on the center of the pathogen lawn and the plates were incubated at 30 °C. The zones of predation were observed with a stereomicroscope (Olympus SZX10, Olympus Corporation, Tokyo, Japan).

### Enzyme production

The sequence of C25GH19B (WP_161664743.1) was analyzed by the tools SignalP (Armenteros et al. 2019) and Phobius (Kall et al. 2007) and was predicted to have a signal peptide (amino acids 1–27). The gene of C25GH19B without signal peptide was codon-optimized (GenBank: OM468604), synthesized, and cloned into the pET-28b vector between the *Nco*I and *Xho*I restriction sites by GENEWIZ (GENEWIZ, Inc., Suzhou, China). The construct was transformed into *E. coli* BL21 (DE3) to express the protein. The resultant *E. coli* BL21 (DE3) cells were cultured at 37 °C and 200 rpm in Luria–Bertani (LB) medium containing kanamycin (50 μg/mL) until the OD_600_ was approximately 0.6. 0.1 mM isopropyl-β-D-thiogalactopyranoside (IPTG) was added and cultured at 22 °C with 200 rpm shaking overnight to induce protein expression. The cells were harvested by centrifugation at 4 °C, and then suspended in lysis buffer (30 mM Tris–HCl, 300 mM NaCl, 1 mM PMSF, 20 mM Imidazole, pH 8.0). After sonication, the cell suspension was centrifuged (4 °C) at 12,000 rpm for 20 min, and the supernatant was subjected to nickel-chelating chromatography. Fractions containing C25GH19B were pooled, concentrated, and dialyzed against 50 mM acetate buffer (pH 5.0) for further analysis.

### Enzyme activity assays

The enzyme activity was determined by the turbidimetric method as previously described (Del Giudice et al. 2013). Briefly, the reaction mixture containing 50 mM acetate buffer (pH 5.0), 0.4 mg/mL purified C25GH19B, 10 mg/mL lyophilized *M. luteus* NCTC2665. The decrease in absorbance at 450 nm was monitored by a Multiskan GO spectrophotometer for 5 min. The control sample contained the buffer instead of the enzyme. The assays were performed in triplicate. The activity was expressed as the rate of decrease in absorbance per minute.

### Effect of pH and temperature on enzyme activity

The optimal pH for enzyme activity was determined by measuring the activity using lyophilized *M. luteus* as the substrate at pH ranging from 3.5 to 8.0. 50 mM citric-phosphate buffer was used for pH 3.5–7.0 and 50 mM phosphate buffer was used for pH 6.5–8.0.

The effect of temperature on the activity of C25GH19B was examined across the range of 25–70 °C by measuring the activity as the above method.

### Thermal inactivation

The purified C25GH19B (1 mg/ml) were incubated at 40 °C and sampled at different intervals to measure the residual activities at 30 °C. The average thermal inactivation rate constants (*k*_*inact*_) were calculated from the plots of ln (residual activity) versus time (Bai et al. 2020). The time required for the residual activity to be reduced to half (*t*_*1/2*_) of the enzymes was calculated by the equation: *t*_*1/2*_ = ln2/ *k*_*inact*_.

The *T*_*50*_^*15*^ value was assayed as previously described (Xie et al. 2014). Briefly, C25GH19B (1 mg/ml) were heated at different temperatures for 15 min, and the residual activities were measured at 30 °C. The residual activity was plotted against temperature and fitted with a sigmoidal curve using Origin 8.0. The *T*_*50*_^*15*^ value is the temperature at which enzyme activity is reduced to 50% after a 15 min heat treatment.

### Effect of metal ions on enzyme activity

As the above-mentioned enzyme activity assays, divalent metal ions (Fe^2+^, Ni^2+^, Cu^2+^, Co^2+^, Ca^2+^, Zn^2+^, Mn^2+^) in the concentration range of 0.1–10 mM were added to the reaction mixture, and the relative activity of C25GH19B (0.2 mg/mL) was determined.

### Bacteriolytic activity of C25GH19B on *C. flaccumfaciens* and *P. syringae*

The bacteriolytic activity of C25GH19B on the pathogen was determined by the turbidimetric method as described above using the lyophilized *Curtobacterium flaccumfaciens* GDMCC 1.343 and *Pseudomonas syringae* GDMCC 1.330 as substrates.

Viable plate counts were also used to test the bacteriolytic activity of C25GH19B on the pathogen. *C. flaccumfaciens* GDMCC 1.343 and *P. syringae* GDMCC 1.330 at the mid-logarithmic phase were washed and resuspended in 50 mM acetate buffer (pH 6.0) containing C25GH19B (0.5 mg/mL). Controls were incubated in the absence of C25GH19B. The mixture was incubated at 30 °C for 2 h, serially diluted, and the dilutions were spread on nutrient agar plates. The colony-forming units (CFU) were obtained after incubating the agar plates at 30 °C for 72 h. All assays were performed in triplicate and the results are the means of three independent experiments.

### Transmission electronic microscopy

*C. flaccumfaciens* GDMCC 1.343 and *P. syringae* GDMCC 1.330 at the mid-logarithmic phase were washed and resuspended in 50 mM acetate buffer (pH 6.0) containing the C25GH19B (0.5 mg/mL). Controls were incubated in the absence of protein. The mixture was incubated at 30 °C for 2 h or 6 h, then the cells were observed using an H7650 TEM microscope (Hitachi, Tokyo, Japan).

### SDS-PAGE analysis of the binding ability of C25GH19B to peptidoglycan

The cell wall peptidoglycan was extracted from *C. flaccumfaciens* GDMCC 1.343 and *P. syringae* GDMCC 1.330, respectively, according to the previously described method (Kim et al. 2020). Each binding reaction contained 10 mg/ml peptidoglycan and 0.5 mg/ml C25GH19B in 50 mM acetate buffer, pH 6.0, and was carried out at 25 °C in a thermomixer set to 1000 rpm. The reaction system for the control group was set in the absence of C25GH19B. After 4 h incubation, the sample was centrifuged at 13,000×*g* for 10 min. The free protein in the supernatant and that bound to the peptidoglycan were analyzed by SDS-PAGE.

### Sequence alignment, phylogenetic analysis, and homology modeling

The protein sequences of the characterized GH19 enzymes were obtained from the CAZy database (http://www.cazy.org/) (Cantarel et al. 2009) and the GH19ED database (https://gh19ed.biocatnet.de) (Orlando et al. 2021). The amino acid sequences were conducted with multiple sequence alignment using the Clustal Omega web server (https://www.ebi.ac.uk/Tools/msa/clustalo/) (Sievers and Higgins 2018). The results were rendered by ESPript 3.0 (Robert and Gouet 2014). The neighbor-joining phylogenetic tree was created by MEGA-X (Kumar et al. 2018), and the figure was generated by the iTOL web server (https://itol.embl.de/) (Letunic and Bork 2019).

The homology model structure of C25GH19B was created by the SWISS-MODEL web server (https://swissmodel.expasy.org/) (Waterhouse et al. 2018) using the crystal structure of the bacteriophage SPN1S endolysin (AEX26904.1, PDB entry: 4OK7) (Park et al. 2014) as the template. Verify_3D (Luthy et al. 1992) was used to check the residue profiles of the 3D models obtained. PROCHECK (Laskowski et al. 1996) analysis was performed to assess the stereochemical qualities of the 3D models. Pymol software (The PyMOL Molecular Graphics System, Version 1.8 Schrödinger, LLC, De Lano Scientific, San Carlos, CA, USA) was used to view the structure and generate figures.

## Results

### *Corallococcus silvisoli* c25j21 can prey on phytopathogenic bacteria

**Fig. 1:**
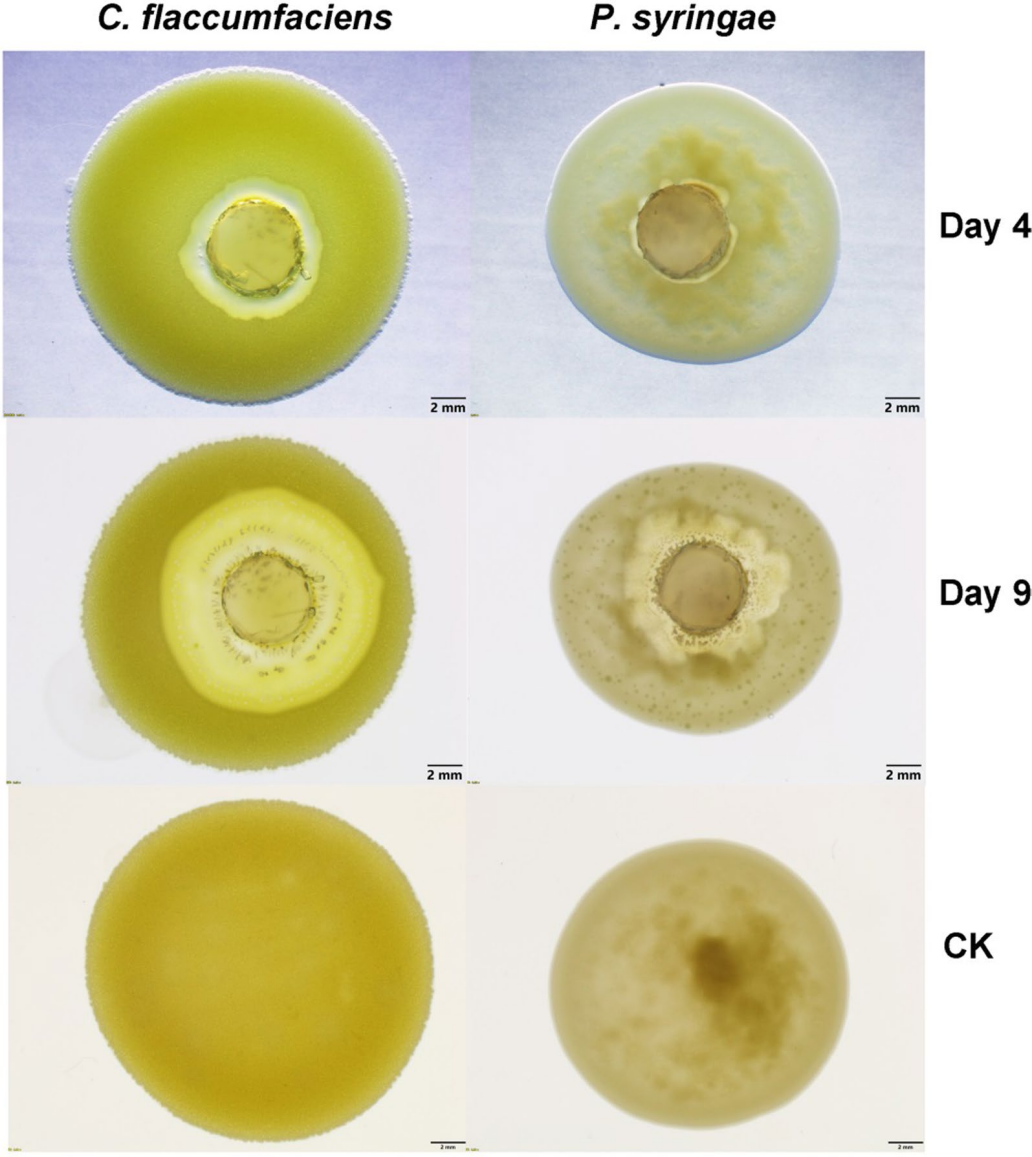
The predation of c25j21 against plant pathogenic bacteria *C. flaccumfaciens* and *P. syringae*

We previously isolated a predatory myxobacterium strain, *Corallococcus silvisoli* c25j21, from soil samples. To investigate whether this strain can prey on phytopathogenic bacteria, we inoculated the agar block with c25j21 onto the lawns of Gram-positive *C. flaccumfaciens* and Gram-negative *P. syringae*, respectively. After 4 days of culture, a lytic zone appeared on the lawns of both *C. flaccumfaciens* and *P. syringae* (Fig. 1). And with the extension of the culture time, the lytic zone gradually expanded. These results indicate that c25j21 can prey on both Gram-positive and Gram-negative phytopathogenic bacteria.

### Mining the bacteriolytic enzymes of c25j21

**Fig. 2:**
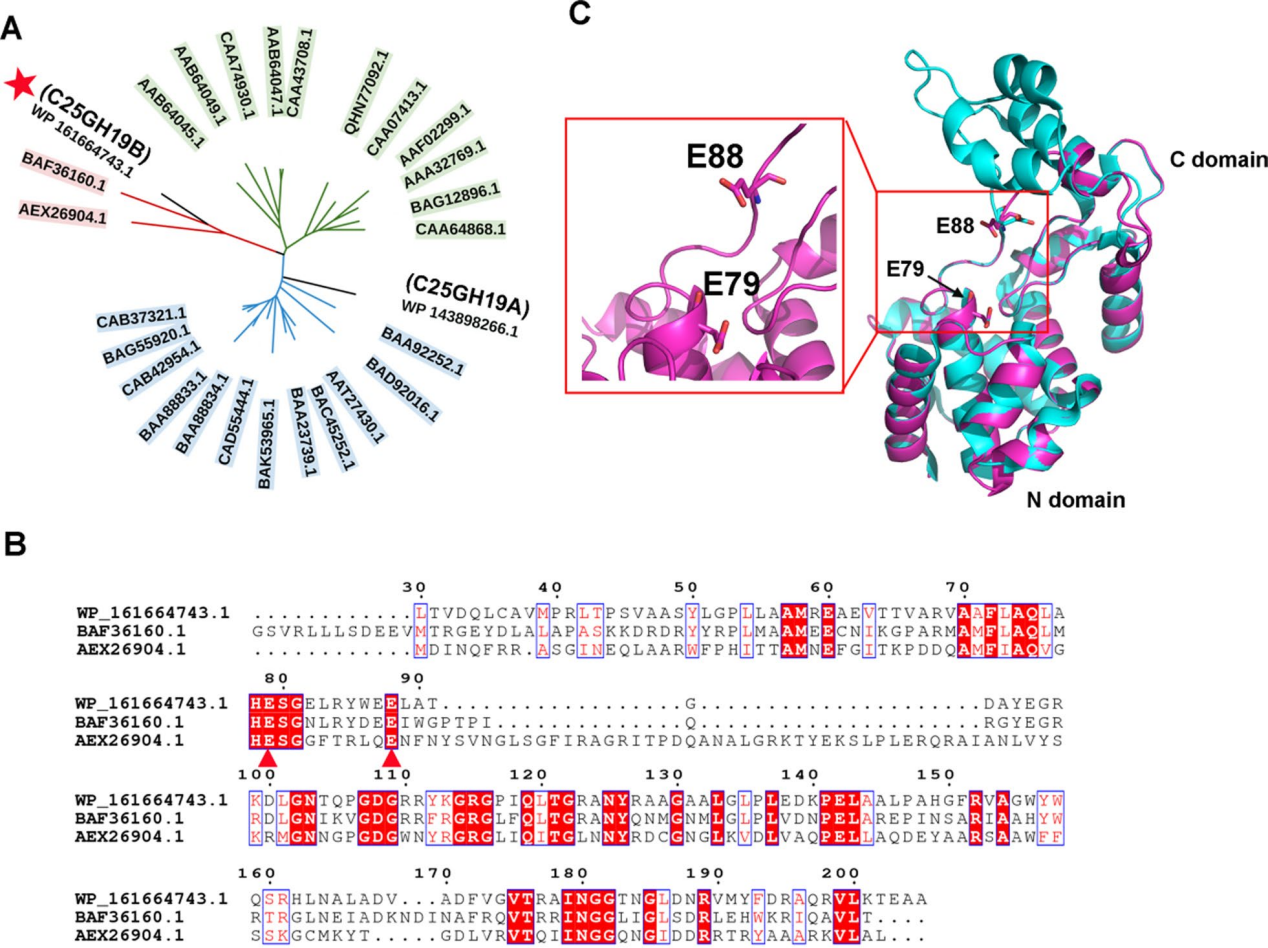
Sequence and structure analysis of C25GH19B. **A** Phylogeny of C25GH19B and the well-characterized GH19 enzymes in the CAZy database. The enzymes derived from plants, bacteria, and bacteriophages are shown in green, blue, and red, respectively. C25GH19B is indicated by a red star. **B** Multiple sequence alignment of C25GH19B and the phage lysozyme (BAF36160.1) and endolysin (AEX26904.1). The catalytic residues are indicated by red triangles. **C** Superposition of the model structure of C25GH19B (magenta) and the crystal structure of AEX26904.1 (cyan, PDB entry: 4OK7). The predicted catalytic residues of C25GH19B are shown in sticks and labeled

To explore the enzymes of c25j21 with the potential antibacterial activity, we analyzed its genome-encoded proteins that might act on the bacterial cell walls. The genome of c25j21 was annotated by NCBI and the dbCAN meta server (Yin et al. 2012). Two proteins, WP_143898266.1 assigned as C25GH19A and WP_161664743.1 assigned as C25GH19B, were annotated as glycoside hydrolase 19 (GH19) family hydrolases. The GH19 family includes chitinases derived from bacteria and plants, and lysozymes or endolysins derived from bacteriophages. Phylogenetic analysis showed that C25GH19A was classified into the bacterial chitinases clade, while C25GH19B was closely related to the phage lysozymes or endolysins (Fig. 2A and Additional file 1: Fig. S1). The sequence identity between C25GH19B and the lysozyme (BAF36160.1) derived from *Microcystis virus* Ma-LMM01 (Hosoda et al. 2011) and the endolysin (AEX26904.1) derived from *Salmonella phage* SPN1S (Park et al. 2014) is 54% and 31%, respectively. According to the multiple sequence alignment (Fig. 2B), the catalytic center of C25GH19B was predicted to be a catalytic diad of E79 and E88, which correspond to the catalytic center E49-E58 of AEX26904.1 (Park et al. 2014).

To analyze the structural characteristics of C25GH19B, we constructed its homology model based on the crystal structure of endolysin AEX26904.1. The model structure was evaluated by PROCHECK and Verify-3D (Additional file 1: Figs. S2 and S3). The model shows that C25GH19B can fold into an endolysin-like three-dimensional structure, with the catalytic center located at the interface of the two domains (Fig. 2C). Similar to BAF36160.1, the C-terminal substrate-binding domain of C25GH19B is smaller than that of AEX26904.1 and lacks three helices (Fig. 2B, C), which may affect their substrate-binding mode (Park et al. 2014; Orlando et al. 2021). Based on the sequence and structure analysis, we speculated that C25GH19B might be an endolysin-like myxobacterial lysozyme.

### Biochemical characterization of C25GH19B

**Fig. 3:**
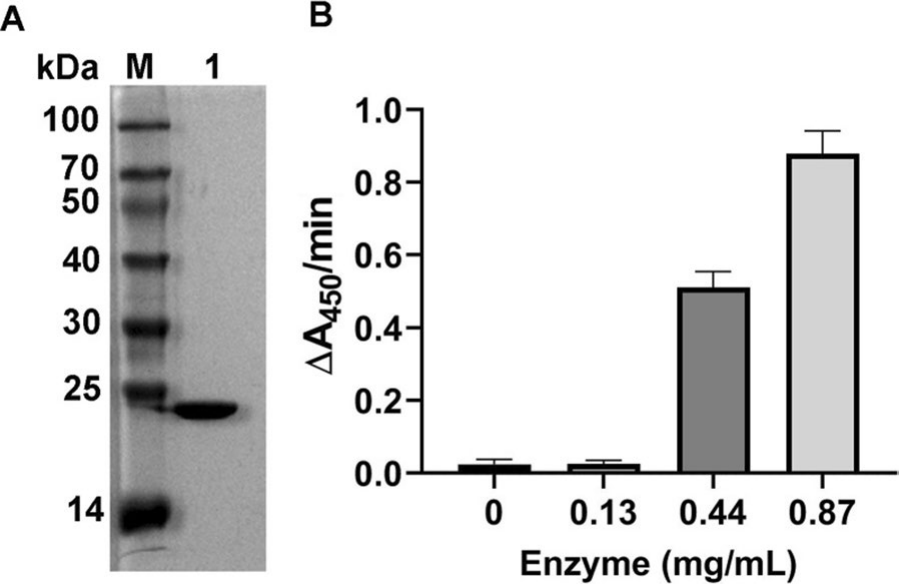
Purification and enzyme activity of C25GH19B. **A** SDS-PAGE analysis of the purified C25GH19B. M, protein molecular weight marker; Lane 1, the purified protein of C25GH19B. **B** The bacteriolytic activity of C25GH19B at different enzyme concentrations determined by using lyophilized *M. luteus* as substrate

The homology of C25GH19B with bacteriophage-derived lysozyme and endolysin encouraged us to explore its bacteriolytic activity. The gene of C25GH19B without the signal peptide was cloned into the pET-28a vector and transformed into *E. coli* BL21(DE3) to express the enzyme protein. The purified C25GH19B was about 20 kDa (Fig. 3A), consistent with the expected molecular weight. Moreover, C25GH19B showed an enzyme concentration-dependent bacteriolytic activity against lyophilized *M. luteus*, indicating that it is a lysozyme (Fig. 3B).

**Fig. 4:**
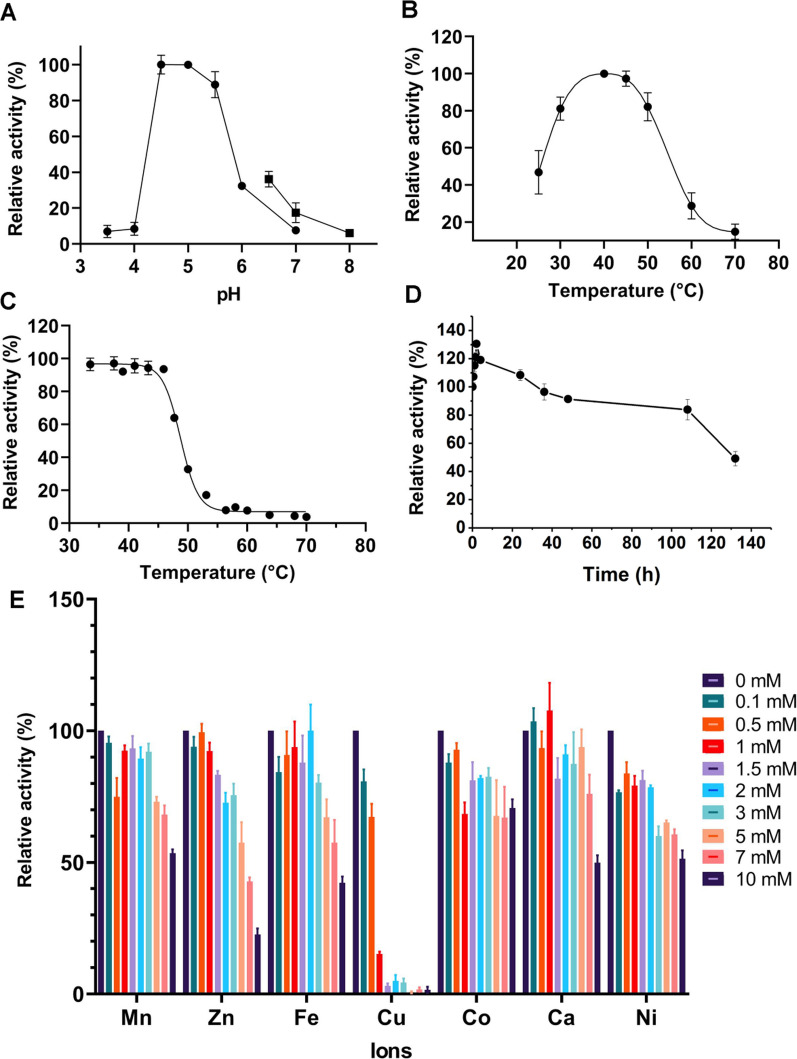
Biochemical characterization of C25GH19B. **A** The pH-activity profile of C25GH19B. The activity was determined in 50 mM citric—phosphate buffer (●) in the range of pH 3.5–7.0 and 50 mM phosphate buffer (■) in the range of pH 6.5–8.0. **B** The temperature-activity profile of C25GH19B. **C** Thermal inactivation of C25GH19B incubated at different temperatures for 15 min. The enzyme protein in 50 mM acetate buffer (pH 5.0) was incubated at various temperatures for 15 min and assayed for residual activity at 30 °C. The activity of the samples without incubation was considered to be 100%. **D** Thermal inactivation profile of C25GH19B at 40 °C. **E** The effect of divalent metal ions on the enzyme activity

To investigate the optimal catalytic reaction conditions of C25GH19B, we first assayed the effect of pH on its activity. As shown in Fig. 4A, C25GH19B was active in the range of pH 3.5–8.0, with an optimum pH of 4.5–5.0. The temperature profile of C25GH19B indicated the optimum temperature was 40 °C and the enzyme activity remained more than 80% in the range of 30 to 50 °C (Fig. 4B). To further characterize the effect of temperature on enzyme activity, we tested the thermal inactivation of C25GH19B. The enzyme was incubated in a temperature range from 35 to 70 °C for 15 min, and the residual activities were measured (Fig. 4C). The *T*_*50*_^*15*^ value, the temperature when half of the enzyme activity is retained after 15 min incubation, was calculated to be 48.7 °C. The 40 °C-thermal inactivation profile showed that more than 50% of the enzyme activity was maintained after 130 h of incubation (Fig. 4D), and the half-life was calculated to be about 155 h. These results indicate that C25GH19B is stable at 40 °C.

We further investigated the effect of divalent metal ions on the enzyme activity of C25GH19B (Fig. 4E). The tested divalent metal ions have no obvious activation effect on C25GH19B but inhibited the enzyme activity at high concentrations, especially Cu(II), which significantly inhibited the enzyme activity when the concentration was up to 0.5 mM, while the enzyme was almost completely inactivated when the concentration of Cu(II) was higher than 1.5 mM.

### Bacteriolytic activity of C25GH19B against phytopathogens

**Fig. 5:**
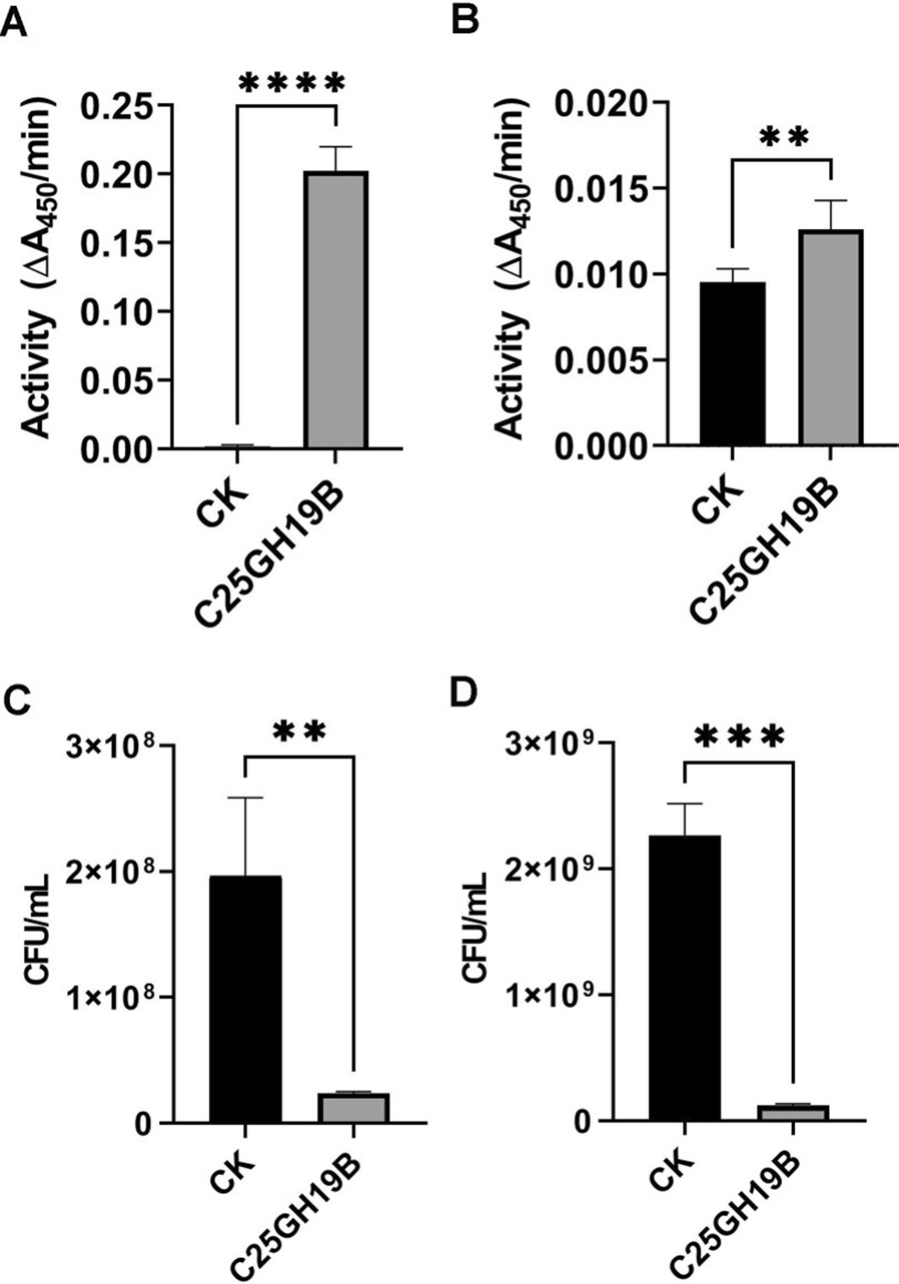
Bacteriolytic activity of C25GH19B against *C. flaccumfaciens* and *P. syringae*. **A** and **B** Lytic activity of C25GH19B on lyophilized *C. flaccumfaciens* and *P. syringae*, respectively. **C** and **D** Plate count assays for the antimicrobial activity of C25GH19B on freshly cultured *C. flaccumfaciens* and *P. syringae*, respectively. **p < 0.01; ***p < 0.001; ****p < 0.0001

Since C25GH19B has lysozyme activity against *M. luteus*, we would like to investigate whether it can lyse plant pathogenic bacteria. *C. flaccumfaciens* and *P. syringae* were used as representatives of Gram-positive and Gram-negative bacteria, respectively, to test the bacteriolytic activity of C25GH19B. As shown in Fig. 5A, C25GH19B showed high lytic activity against *C. flaccumfaciens*, which is consistent with its lytic activity against *M. luteus*, indicating that the enzyme has a bacteriolytic activity on Gram-positive bacteria. In addition, although slightly less active, C25GH19B showed lytic activity against *P. syringae* (Fig. 5B), suggesting that this enzyme was also active on Gram-negative bacteria.

**Fig. 6:**
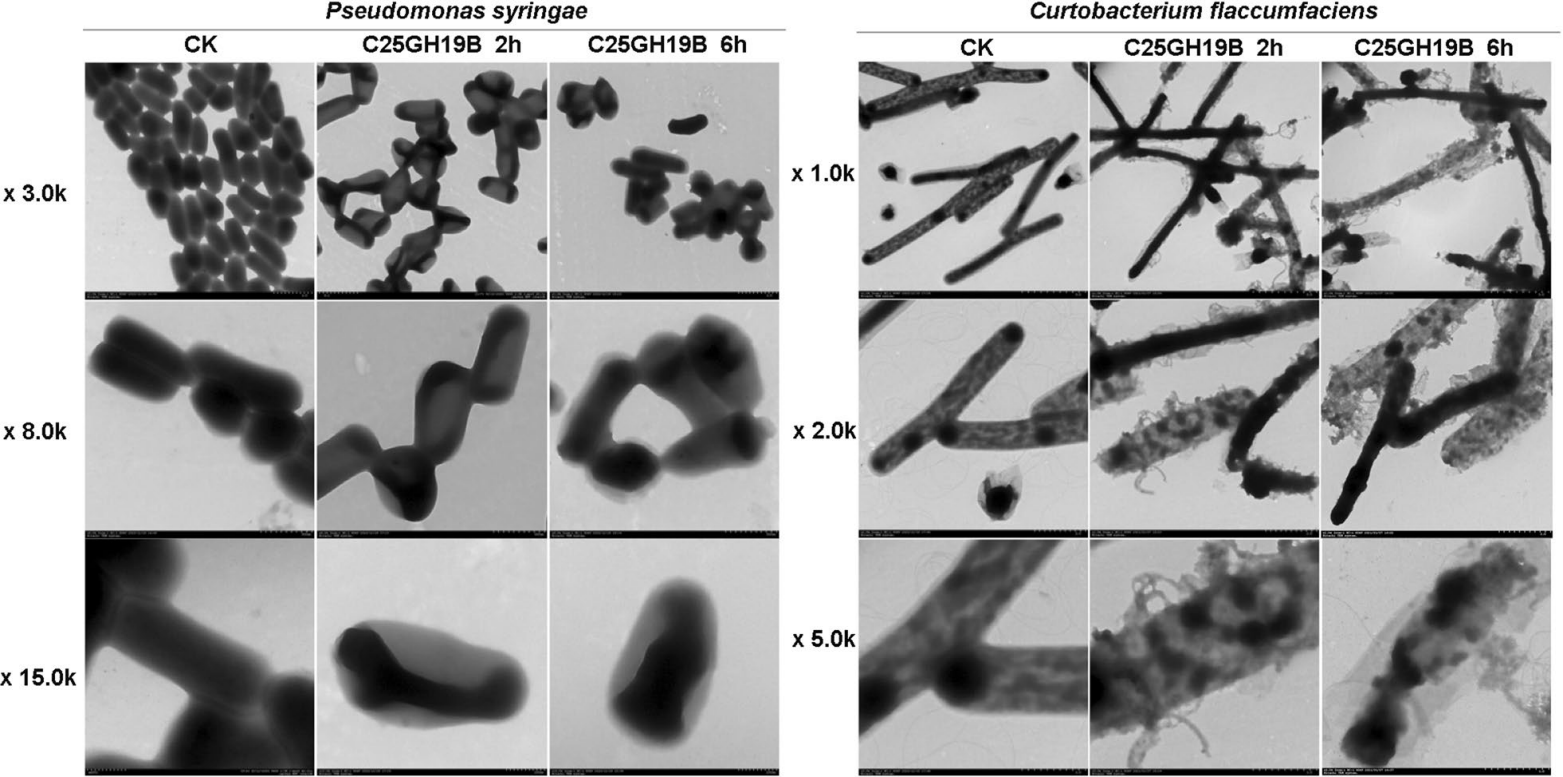
Transmission electron microscopy of *C. flaccumfaciens* and *P. syringae*. The freshly cultured cells were treated with 0.5 mg/mL C25GH19B at 37 °C for 2 h or 6 h. 50 mM sodium acetate buffer was used as the control group

The antimicrobial activity of C25GH19B was further tested using plate count assays (Fig. 5C, D). Treated with 0.5 mg/mL C25GH19B for 2 h, freshly cultured *C. flaccumfaciens* and *P. syringae* cells were killed by 88% and 95%, respectively. Transmission electron microscopy (Fig. 6) showed the disruption of the cell wall of *C. flaccumfaciens* treated by C25GH19B. While *P. syringae* treated with C25GH19B showed morphological changes and loss of cytoplasmic contents. Moreover, the longer the treatment time, the more obvious the bacteriolytic effect. Altogether, these results suggest bacteriolytic activity of C25GH19B against both Gram-positive and negative phytopathogens.

**Fig. 7:**
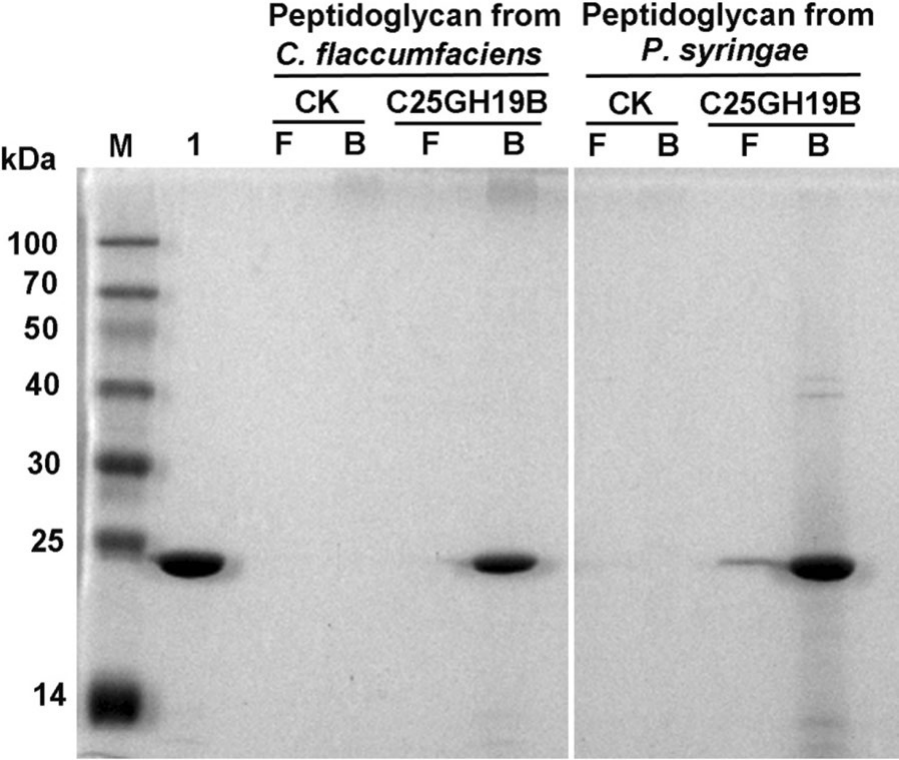
SDS-PAGE analysis of the binding ability of C25GH19B on phytopathogenic bacterial peptidoglycan. Lane M, protein molecular weight marker; Lane 1, free C25GH19B protein; CK, peptidoglycan treated with the acetate buffer; C25GH19B, peptidoglycan treated with C25GH19B; F, free protein in the supernatant; B, peptidoglycan-bound protein

C25GH19B showed bacteriolytic activity against *M. luteus* and phytopathogenic bacteria. We speculated that it might act on peptidoglycan of the bacterial cell wall. To test this speculation, we studied its binding ability to the cell wall peptidoglycan of *C. flaccumfaciens* and *P. syringae*. As shown in Fig. 7, C25GH19B could bind well to the peptidoglycans from the two pathogens, indicating that the target of C25GH19B acting on pathogens is the cell wall peptidoglycans.

## Discussion

*C. silvisoli* c25j21 is a soil indigenous myxobacterium strain that preys on a variety of plant pathogens and could be a potential biocontrol agent. A GH19 family enzyme derived from c25j21, C25GH19B, was characterized in this study. Sequence analysis revealed that C25GH19B is homologous to bacteriophage-derived endolysin and lysozyme, but not bacterial chitinases. Biochemical characterization showed that C25GH19B had peptidoglycan-hydrolysis but not chitin-hydrolysis activity, which further confirmed that it was a myxobacterial lysozyme. C25GH19B prefers a weakly acidic environment with an optimal pH of 4.5–5.0, which is different from the previously reported endolysins, such as LYS177 and LYS188, whose optimal pH is 6–7 (Orlando et al. 2020), while SPN1S endolysin prefers an alkaline environment with an optimal pH of 9.5 (Lim et al. 2012). These enzymes with different physicochemical properties provide diverse candidate molecules for the development of antibacterial agents with different needs.

Unlike LlpM from *M. xanthus*, which only lyses Gram-positive bacteria (Arend et al. 2020), C25GH19B exhibits bacteriolytic activity against both Gram-positive and Gram-negative phytopathogens, e.g. *C. flaccumfaciens* and *P. syringae*. Due to the outer membrane barrier of Gram-negative bacteria, the enzyme does not easily access its peptidoglycan, which is reflected in the lower activity of C25GH19B against *P. syringae* when measured by turbidimetry. However, the partial destruction of the cell wall can cause leakage of the cell contents and kill the bacteria, so we could see that C25GH19B also has good bacteriolytic activity against Gram-negative bacteria *P. syringae* in the plate counting experiment and scanning electron microscope observation. The results of the substrate binding experiments indicated that C25GH19B acts on the cell wall peptidoglycan of phytopathogenic bacteria. These results suggested that C25GH19B has the potential to be developed as a component of novel biocontrol agents.

Myxobacteria have a broad predatory spectrum, but most of them have not been well studied except for the model organism *M. xanthus* (Thiery and Kaimer 2020). Microbial predation is a complex process that may involve a variety of enzymes and other components, which provides abundant resources for discovering new biocontrol agents. Further exploration and characterization of the enzymes of myxobacteria, especially those less-studied strains such as *Corallococcus* sp., *Vitiosangium* sp., and *Pyxidicoccus* sp., etc., are of great significance for the development of novel biocontrol strategies.

In summary, we characterized the GH19 enzyme, C25GH19B, from the myxobacterium strain *C. silvisoli* c25j21. C25GH19B is an endolysin-like enzyme with bacteriolytic activity against both Gram-positive and Gram-negative phytopathogens. Our results provide an experimental basis for the development of myxobacterial extracellular enzymes as novel biocontrol agents.

## Supplementary Information

**Additional file 1: Figure S1.** Phylogeny of C25GH19B and the GH19 proteins in the GH19ED database. The members of the chitinases and endolysins sub-families in GH19ED database are shown in blue and red, respectively. **Figure S2.** Ramachandran plot of the structure model of C25GH19B. **Figure S3.** Verify_3D evaluation of the structure model of C25GH19B.

## Data Availability

All data generated or analyzed during this study are included in this manuscript.
