# Supplementary material for: A myxobacterial GH19 lysozyme with bacteriolytic activity on both Gram-positive and negative phytopathogens
Source: AMB Express. 2022 May 12;12:54. doi: 10.1186/s13568-022-01393-y (PMC9098779; doi:10.1186/s13568-022-01393-y)
Supplement: Supplementary file 1 — Additional file 1: Figure S1. Phylogeny of C25GH19B and the GH19 proteins in the GH19ED database. The members of the chitinases and endolysins sub-families in GH19ED database are shown in blue and red, respectively. Figure S2. Ramachandran plot of the structure model of C25GH19B. Figure S3. Verify_3D evaluation of the structure model of C25GH19B. [file 13568_2022_1393_MOESM1_ESM.docx]

**A myxobacterial GH19 lysozyme with bacteriolytic activity on both Gram-positive and negative phytopathogens**

**Additional Figures**

**
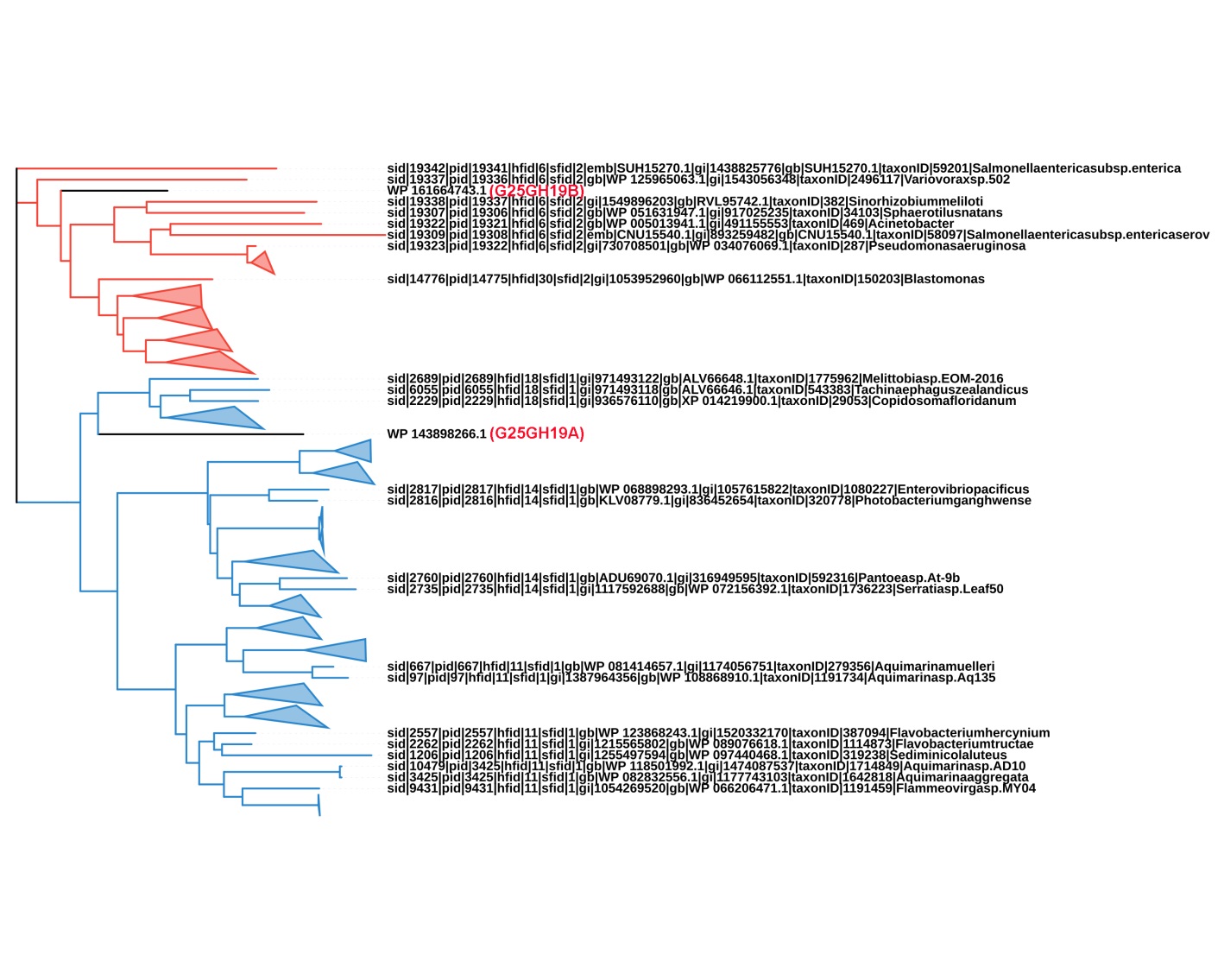
**

**Figure S1** Phylogeny of C25GH19B and the GH19 proteins in the GH19ED database. The members of the chitinases and endolysins sub-families in GH19ED database are shown in blue and red, respectively.


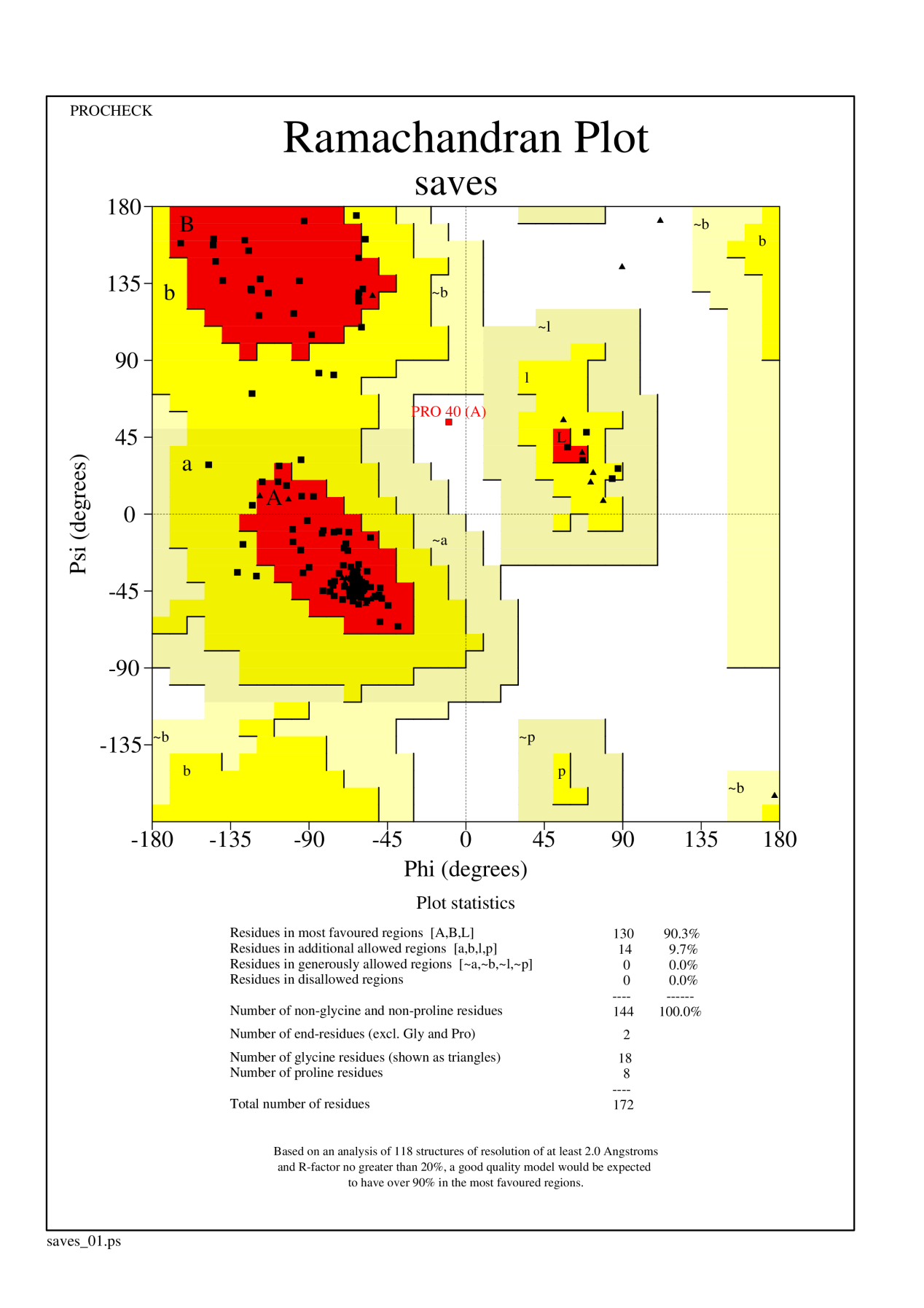


**Figure S2** Ramachandran plot of the structure model of C25GH19B.


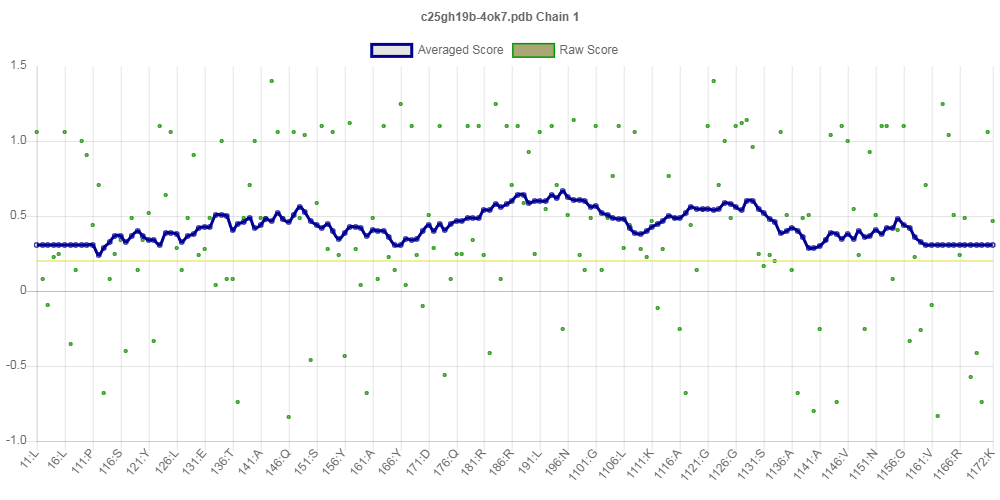


**Figure S3** Verify_3D evaluation of the structure model of C25GH19B.
